# Design and Additive Manufacturing of 3D Phononic Band Gap Structures Based on Gradient Based Optimization

**DOI:** 10.3390/ma10101125

**Published:** 2017-09-22

**Authors:** Maximilian Wormser, Fabian Wein, Michael Stingl, Carolin Körner

**Affiliations:** 1 Joint Institute of Advanced Materials and Processes (ZMP), Friedrich-Alexander-University Erlangen-Nürnberg, 90762 Fürth, Germany; 2 Department Mathematik, Mathematical Optimization, Friedrich-Alexander-University Erlangen-Nürnberg, 90762 Fürth, Germany; fabian.wein@fau.de (F.W.); michael.stingl@fau.de (M.S.); 3 Materials Science and Technology for Metals (WTM), Friedrich-Alexander-University Erlangen-Nürnberg, 90762 Fürth, Germany; carolin.koerner@fau.de

**Keywords:** phononic band gap, additive manufacturing, selective electron beam melting, cellular materials, metamaterials, topology optimization, parametric shape optimization, gradient based optimization

## Abstract

We present a novel approach for gradient based maximization of phononic band gaps. The approach is a geometry projection method combining parametric shape optimization with density based topology optimization. By this approach, we obtain, in a two dimension setting, cellular structures exhibiting relative and normalized band gaps of more than 8 and 1.6, respectively. The controlling parameter is the minimal strut size, which also corresponds with the obtained stiffness of the structure. The resulting design principle is manually interpreted into a three dimensional structure from which cellular metal samples are fabricated by selective electron beam melting. Frequency response diagrams experimentally verify the numerically determined phononic band gaps of the structures. The resulting structures have band gaps down to the audible frequency range, qualifying the structures for an application in noise isolation.

## 1. Introduction

In the last 25 years since their emergence, materials with phononic band gap (PBG) are gaining more interest among scientists. A PBG describes a frequency band where mechanical waves are not transmitted through a material. For applying the concept of PBGs in real life, the need for design methods to precisely predict the resulting PBG frequency range arises. Furthermore, for a possible application as a sound isolator or filter, the PBG must lie within the audible frequency range of 20 Hz to 20 kHz [1].

PBGs can occur in many kinds of composite structures. Phononic crystals, named based on the previously discovered photonic crystals, are periodic binary composites and were the first structures identified to have a PBG [1]. These composites consist of periodic inclusions that act as scatterers and a matrix with a large mismatch in elastic constants compared to the inclusions [2]. While the first numerical proof of this concept goes back to 1993 [3], the first experimentally observed PBG in a phononic crystal was documented in 1995 and a complete PBG was first documented in 1998 [4,5]. A complete PBG is defined as a band gap that is independent of wave polarization or travel direction [1]. Only a few years later, a new type of PBG material where the wave length is much smaller than the lattice constant of the periodic array was discovered. These are called sonic crystals and are a type of acoustic metamaterial that relies on locally resonant structures in order to form a PBG in very low frequencies [6,7]. A good description of these and additional PBG material types is given in [8].

A wide range of different approaches on how to design band gap structures has been realized in the past. Most of these are 2D structures, e.g., planar grid structures with tunable non-structural masses [9,10], tetragonal and hexagonal chiral lattices [11,12], auxetic inverted honeycombs [13], or modified honeycomb lattices [14]. In three dimensions, cubic lattices with spherical holes [15,16], sinusoidal beams [17], octahedrons using inertial amplification mechanisms [18], and cubic lattices found by eigenmode analysis of regular lattices [19] are some examples for the geometry of PBG materials. Research following the eigenmode approach showed that sinusoidal strut lattices, e.g., from eigenmode shapes of regular cubic unit cells, have favorable PBG properties [20,21]. It was also found that the eigenmode shape with the most favorable PBG properties shows auxetic behaviour in all Cartesian coordinate directions [19].

**Figure 1:**
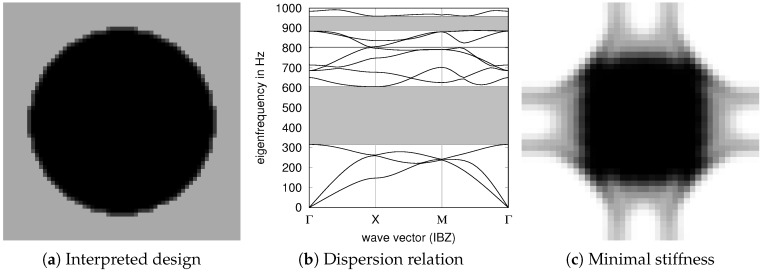
(**a**) The periodic 2D design is an interpretation of the optimal design obtained in [22]. The solid inclusion is modeled by multiplying the local material with a pseudo density value ρ=1 (black) while the weak matrix material is modeled by multiplying the material with ρ=0.1 (dark gray); (**b**) shows the dispersion relation corresponding to (**a**); (**c**) To get rid of the weak matrix material in (**a**), the lower bound of the pseudo density is reduced to $1\times 10^{-9}$ (white), corresponding to air. Without a soft matrix material to carry the solid mass, a structural connection for the periodic structure is necessary. The figure shows the obtained optimization result with concurrent stiffness constraints for the structure. The different levels of grayness correspond to graded stiffness of the material which is not manufacturable. See the discussion in Section 2.2.

PBGs have been also designed by mathematical programming. In the landmark paper [22], Sigmund and Jensen proposed a density based topology optimization approach to optimize for PBGs. A periodic bi-material design with a stiff inclusion within a weaker but existing matrix material is assumed (see Figure 1a and the corresponding band gap in Figure 1b). We discuss and extend the approach in Section 2.2. The band gap of the design in Figure 1a can easily be enlarged by increasing the contrast of stiffness and mass between the solid inclusion and the matrix material. The nonphysical extreme case is solid material placed freely within air with obviously no transmission at all.

For practical reasons, a connected lattice design is desired without a weak matrix material to carry the solid structure. While density based topology optimization, from [23,24], can efficiently be applied for the design of lattice structures, the combination of PBG maximization and concurrent connectivity/stiffness constraints is difficult to realize, see Figure 1c. PBG optimization problems are very sensitive with respect to intermediate materials [25]. To this end, far less efficient gradient free optimization has been applied for PBG optimization of connected structures [26,27,28]. With reformulated connectivity constraints, the realization of connected PBG optimization by gradient based topology optimization was successfully shown [29].

Considering the issue of nonphysical design and unconnected structures is crucial for the optimization process, we propose a novel optimization approach with a reduced design space where both issues are under full control of the problem formulation but not any more subject for the optimizer to change. This basically means that there is a rigorous formulation for the minimal strut thickness.

In this research, we present novel PBG structures obtained by our new optimization approach. As the optimization of 3D PBG structures with our approach is not yet available, we deduce common design patterns from the 2D optimization results and extend them to create a novel 3D lattice design. Samples from these new unit cells are fabricated from metal powder by selective electron beam melting. The numerical results are compared to experimental results from a transmission experiment.

## 2. Methods

### 2.1. Dispersion Relation

Dispersion describes the phenomenon that the relation of the phase velocity $v_{p}$ of a wave in a medium and its frequency ω is no longer linear. Therefore, the dispersion relation relates the wave vector *k* to the frequency ω and the phase velocity $v_{p}$ as follows [30].

(1) $$v_{p}\left(\omega \right)=\frac{\omega }{k(\omega )}$$

**Figure 2:**
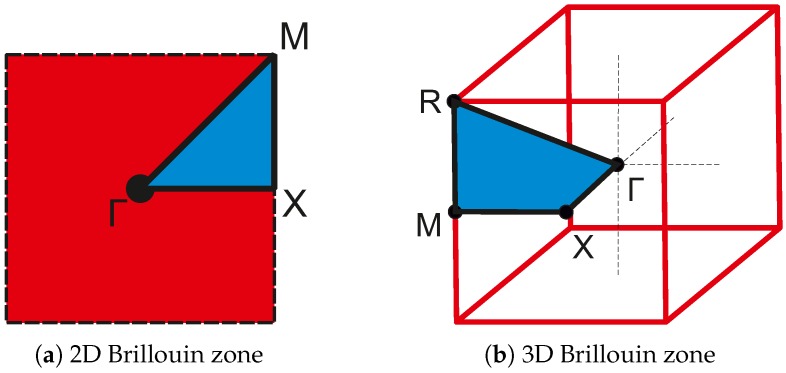
Brillouin zones (red) and irreducible Brillouin zones (IBZ, blue) for the (**a**) two-dimensional case and (**b**) three-dimensional case.

According to the Bloch theorem, the wave propagation behavior of a periodic lattice can be fully described by just analyzing its unit cell [12,31]. The relation of *k* and the phase velocity $v_{p}$ is a linear eigenvalue problem that has to be solved for all possible values of *k* in order to fully characterize the unit cell. The path on which the values of ω have to be calculated is given by the first Brillouin zone, which is the Wigner-Seitz cell of the reciprocal lattice [31]. The first Brillouin zone can be further reduced to the irreducible Brillouin zone (IBZ), whose complete path along its vertices is used to calculate the dispersion relation. In a quadratic lattice this path is Γ–X–M–Γ; in a cubic lattice the path is extended by one additional point to Γ–X–M–R–Γ (see Figure 2). By calculating the eigenvalues of ω for values of *k* along this path, we get the dispersion relation, as can be seen in Figure 1b. The regions without any possible ω in any direction of *k* are called complete band gaps and are from here on referred to simply as PBG, see also Figure 1b.

The 2D dispersion relations are obtained by an in-house academic software, while 3D dispersion relations are calculated with the commercial finite element software COMSOL Multiphysics 5.3. A strut thickness of 0.5 mm was assumed for the 3D structures based on the estimated mechanically active diameter of the actual samples (see Section 2.3). Material parameters for Ti-6Al-4V (ρ = 4420 $kg/m^{-3}$, *E* = 114 GPa, nu = 0.33) for the 3D simulations are taken from the included materials library. Floquet-periodicity is applied to the opposing strut ends of the unit cell.

### 2.2. Gradient Based Structural Optimization

Structural optimization has become widely accepted as a design tool in academic research and industry. Two prominent classes are topology and shape optimization. Density based topology optimization allows the creation of an arbitrary number of holes with arbitrary shapes. Thus, very complex designs of high performance can develop through the optimization process while typically not much preparatory work is necessary to set up the optimization problem including the definition and initial design of the design domain. The idea of topology optimization is to introduce *pseudo density* variables $\rho_{e}\in \left(0,1\right]$ with cell index *e* for each mesh cell to scale the local material element-wise constant from solid material (1) to void (a very small value) [23,24], see also Figure 1.

By extending the density based PBG problem [22] to a solid and void ($\rho_{\min}=1\times 10^{-9}$) problem and adding a stiffness condition [32] to obtain connectivity, we obtain the solution shown in Figure 1c. The black region is to be interpreted as solid material, the white region with $\rho_{\min}$ is to be interpreted as void. However, the regions with intermediate pseudo density (also called *gray* regions) have no straight physical interpretation and therefore the design has no practical value. Having potentially non-interpretable designs due to intermediate pseudo density is one of the drawbacks of density based topology optimization. For the majority of common applications there are approaches to overcome this issue (e.g., Heaviside projection based approaches [33,34]). However, for the present problem we were not able to apply the Heaviside projection successfully. Heaviside projection is based on a smoothed step function where the smoothing parameter is gradually increased towards a steeper function by means of successive optimization problems starting from the previous result. In our experiments, the optimizer was not able satisfy the PBG and stiffness constraints concurrently when the smoothing parameter was increased to moderate value. This might be due to an increasing non-differentiability in combination with the per se difficult nonlinear constraints.

To this end, we developed a parametric shape optimization approach which allows us to explicitly formulate the connectivity requirement as given, with no design freedom at hand for the optimizer to remove the connectivity for the sake of PBG maximization. To overcome classical issues of shape optimization like remeshing, we project the geometry on a pseudo density field as in density based topology optimization. With this projection we have full control on the grayness of the interface. We present the details of the method itself in [35] but give a brief introduction in the following. The principal idea of geometry projection can be also found in [36,37], where a set of geometric primitives is moved and scaled.

#### 2.2.1. Shape Mapping

**Figure 3:**
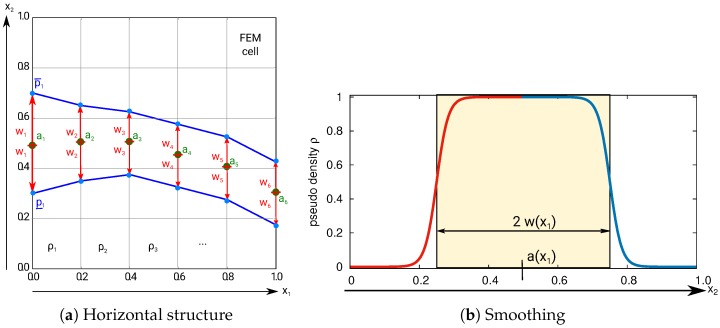
(**a**) An exemplary horizontal structure constructed by the shape variables $a_{1}$, … $a_{6}$ for the height of the center nodes and profile variables $w_{1}$, …$w_{6}$; (**b**) The discrete structure is smoothed by the tanh based function $t_{\beta }$ (2). The parameters $a(x_{1})$ and $w(x_{1})$ are obtained by linear interpolations of the discrete design variables, see (**a**). The smoothing function corresponds to the vertical cross section of the horizontal structure in (**a**).

We start with the introduction of the design variables. Consider a simple horizontal structure as depicted in Figure 3a. There are center variables $a_{1}$, …, $a_{6}$. These variables are the vertical positions of the points $a_{i}$ with 1≤i≤6. The horizontal components of $a_{i}$ are fixed. Associated to the center variables $a_{i}$ are the profile variables $w_{i}$. The structure is constructed by the points ${\bar{p}}_{i}$ and ${\underline{p}}_{i}$ where the vertical positions are constructed as ${\bar{p}}_{i}=a_{i}+w_{i}$ and ${\underline{p}}_{i}=a_{i}-w_{i}$. The horizontal distribution of the variables is directly connected to the finite element discretization. The surface of the structure is a linear interpolation of the points ${\bar{p}}_{i}$ and ${\underline{p}}_{i}$.

This example demonstrates that there is a mandatory vertical connection given *by design*. We just need to make sure that the horizontal structure does not become too thin by design bounds $w_{i}\geq W_{-}^{*}$ (13). Even with a given minimal thickness, the optimizer could try to weaken the horizontal stiffness by designing a zigzagged structure. This is prevented by local linear slope constraints (9) and curvature constraints (10) and (11). See also Section 3.1 for further details.

The structure is now mapped to a *pseudo density* field ρ where a pseudo density value $\rho_{e}$ is assigned to each finite element cell. When a cell is completely covered by the structure, the pseudo density value is one, if it is not covered by the structure the value is very small (here $1\times 10^{-6})$ to represent void. Partially covered elements are explained below. For the finite element analysis the local stiffness and mass matrices are scaled by the pseudo density value. The parametrization by of the stiffness and mass matrix by pseudo density is exactly the standard density based topology optimization approach, also known as SIMP model, see [24]. This leads to the state problem formulation (7). Also the established sensitivity analysis of functions with respect to ρ can be reused. The only necessary step is to have a differentiable mapping from the center variables a and profile variables w to the pseudo density field ρ.

To this end, the boundary of the structure is smoothed by a hyperbolic tangent function, see Figure 3b,

(2) $$t_{\beta }\left(a\left(x_{1}\right),w\left(x_{1}\right),x_{2},\beta \right)=\left\{\begin{array}{cc} 1-\frac{1}{e^{\beta (x_{2}-a\left(x_{1}\right)+w\left(x_{1}\right))}+1} & \mathrm{if}\;x_{2}\leq a\left(x_{1}\right)\\ \frac{1}{e^{\beta (x_{2}-a\left(x_{1}\right)-w\left(x_{1}\right))}+1} & \mathrm{else} \end{array}\right.$$

Here β is the smoothing parameter and $a(x_{1})$ and $w(w_{1})$ are linearly interpolated between the neighboring discrete variables from the set of vectors a and w. A single pseudo density value is obtained by the mapping function, see (8),

(3) $$T_{e}\left(a,w,\beta \right)=\rho_{\min}+\left(1-\rho_{\min}\right)\;\int_{\Omega_{e}}t_{\beta }\left(a\left(x_{1}\right),w\left(x_{1}\right),x_{2},\beta \right)\;d\left(x_{1},x_{2}\right)$$

**Figure 4:**
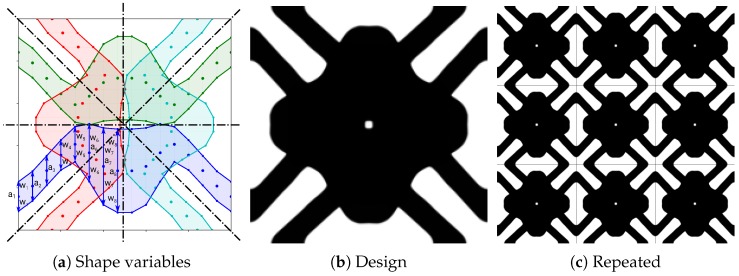
An exemplary result of the phononic band gap optimization is shown: (**a**) Shape variables (coarsened for presentation) of a square symmetric design; (**b**) The structures are mapped to high contrast pseudo density with a very small gray interface; (**c**) Repeated design.

The integration in (3) is performed numerically. Finite element cells only partially covered by the structure result in an intermediate pseudo density, also called *gray* cells. Due to the smoothing by $t_{\beta }$ also the neighboring cells might be gray, depending on β. For our results shown in Figure 4 and Section 3.1 the resolution of the finite element mesh is sufficiently fine and β is large enough to make sure that the smoothed boundary is of no issue (numerically validated by a binary design).

#### 2.2.2. PBG Problem Formulation

For the PBG problem, the left side of the lower horizontal strut (blue in Figure 4a) is parametrized by vertical center positions $a_{i}$ and profile variables $w_{i}$. To respect the square symmetry requirement for reduced Bloch-Floquet analysis on the IBZ zone, the left part of the blue structure is vertically mirrored. The full blue structure is horizontally mirrored to the green structure. The red and cyan structures are obtained by diagonal mirroring.

Overlapping of structures is realized by summing their pseudo density values, however, we still require the resulting pseudo density to not be larger than one. For gradient based optimization min(∑structures,1) needs to be differentiable. For the sake of brevity we refer to [35] for the specific choice and an elaborately discussion of the issue.

We can combine the sensitivity of PBG optimization with respect to pseudo density as in [22] via chain rule with the sensitivity of the linear interpolation of the values, the smooth structure mapping $t_{\beta }$, the integration $T_{e}$ and the smoothing of the overlapping. We formulate the following optimization problem which can be solved by any first order optimization algorithm (we choose SNOPT (Sparse Nonlinear OPTimizer, a commercial software package for solving large-scale nonlinear optimization problems) [38])

(4) $$\begin{array}{cc} \max_{a,w,\alpha ,\gamma } & \frac{2\;\gamma }{\alpha } \end{array}$$

(5) $$\begin{array}{ccc} s.t.\;\;\omega_{jl} & \leq & \alpha -\gamma ,\;1\leq j\leq 6,\;1\leq l\leq 3 \end{array}$$

(6) $$\begin{array}{ccc} \omega_{jl} & \geq & \alpha +\gamma ,\;1\leq j\leq 6,\;4\leq l\leq 12 \end{array}$$

(7) $$\begin{array}{ccc} \left(K\left(k_{j},\rho \right)-\omega_{jl}^{2}M\left(\rho \right)\right)\Phi_{jl} & = & 0,\;\;1\leq j\leq 6,\;1\leq l\leq 12 \end{array}$$

(8) $$\begin{array}{ccc} \rho_{e} & = & T_{e}\left(a,w,\beta \right) \end{array}$$

(9) $$\begin{array}{ccc} |a_{i}-a_{i+1}| & \leq & 1.1/N \end{array}$$

(10) $$\begin{array}{ccc} |a_{i-1}-2\;a_{i}+a_{i+1}| & \leq & c*/N \end{array}$$

(11) $$\begin{array}{ccc} |w_{i-1}-2\;w_{i}+w_{i+1}| & \leq & c*/N \end{array}$$

(12) $$\begin{array}{ccc} a_{i} & \in & [0,0.5],\;1\leq i\leq N/2 \end{array}$$

(13) $$\begin{array}{ccc} w_{i} & \in & [W_{-}*,0.2],\;1\leq i\leq N/2 \end{array}$$

The problem formulation includes the state problems (7), which are to be fulfilled implicitly, which means to be solved within each iteration to provide eigenfrequencies $\omega_{jl}$ and eigenmodes $\Phi_{jl}$. To this end, (7) needs to be solved 72 times within each iteration; for six wave vectors $k_{j}$ to sample the IBZ, we obtain the first 12 eigenfrequencies $\omega_{jl}$ and eigenmodes $\Phi_{jl}$ for each wave vector.

The optimization problem is formulated in terms of vectors of shape variables a and w and two auxiliary variables α and γ. The center frequency of the gap is expressed as α and the width of the gap by 2γ. The cost function (4) maximizes the normalized band gap (size of the gap divided by center frequency). The auxiliary variables are found such that the first three eigenfrequencies are below the lower gap (5) for all wave vectors and all higher eigenfrequencies are above the upper gap (6). With this formulation, we try to cope with two well known non-smoothness issues of eigenfrequency optimization: multiple eigenfrequencies and mode switching (both can be seen in Figure 1b).

We assume a regular discretization of a quadratic 1 m × 1 m finite element (FE) mesh by $N^{2}$ rectangles (here N=200). Each of the $N^{2}$ corresponding pseudo densities $\rho_{e}$ depends on the shape variables a, w and smoothness parameter β via the mapping $T_{e}$ (8), see Section 2.2.1. Like the state Equations (7), the mapping is implicit and not provided explicitly in form of equality constraints to the optimizer.

The Equations (9)–(11) regularize the problem. By regularization, the design space is limited, either to exclude undesired designs and/or to prevent that the optimization algorithm gets locked in poor local optima. With our specific design parametrization, we get strict control of the design. The regularization constraints are local constraints. There are approximately N/2 linear constraints for each constraint formulation above. By (9) the slope of the center line of the strut is controlled. The horizontal spacing from $a_{i}$ to $a_{i+1}$ is 1/N. Restricting the maximal vertical difference to 1.1/N allows just a little more than a 45° ascent or descent. We can restrict the minimal structural thickness to $\sqrt{2}\;W_{-}^{*}$ (The minimal thickness (twice the profile variable *w*) of a horizontal structure is $2\;W_{-}^{*}$, for a 45° angle the minimal thickness reduces to $1/\sqrt{2}$), otherwise the optimizer would form a very thin highly oscillating vertical connection of low stiffness. However, even with the slope constraints, the optimizer could form a zigzag shape. With curvature constraints on both the center line (10) and profile (11) we control the radius of shape features.

In (12), we restrict the horizontal structure to the lower left square and in (13) the design space for the corresponding profiles is set with a lower bound $W_{-}^{*}$ as driving parameter and a sufficiently large upper value. For the sake of clarity, we omit the symmetry mirror operations explained above and shown in Figure 4a and refer to [35].

### 2.3. Sample Fabrication and Measurement Setup

The selective electron beam melting (SEBM) process enables the production of very complex parts, such as cellular metals [39]. It has been shown that the process is suited to fabricate cellular auxetic structures with the ability to control relative density [40,41,42,43]. Furthermore, phononic band gap structures were produced by SEBM and the existence of the complete PBGs was already experimentally verified [21,44].

The cellular metal samples were fabricated by SEBM, a powder-based additive manufacturing technique that utilizes an electron beam for melting powder particles into a dense part in a layer-by-layer fashion [39]. The SEBM machine used for producing the samples is an Arcam AB Q10 and the powder is Ti-6Al-4V with a particle size distribution between 45 μm and 105 μm. All samples have been fabricated with the same machine parameters, consequently producing the same strut thicknesses. The structures produced by SEBM have an inherent surface roughness because of the surrounding powder that gets partially molten and sticks to the surface of the parts. Based on experience with the parameter set, we expect the samples to be dense (less than 0.5 % porosity) and therefore interaction effects with pores are negligible.

The 2D optimization results described in Section 3.1 were used to identify principles to create an improved 3D unit cell geometry. The overall design concept was to use cuboid masses and connect them with weakened struts, in this case of sinusoidal shape inspired by previous works [19,20]. In these works, struts that were rotated outwards by 45° proved as favorable configuration for strut-based unit cells. The straight struts with 90° bends from the optimization results are unfavorable for the SEBM process, as the melt lines differ widely depending on the orientation (horizontal or vertical). This could lead to different strut thicknesses, while the sinusoidal shape can guarantee a relatively constant melt line length regardless of the orientation. Thus, the combination of the masses emerging from the 2D optimization with the already known sinusoidal struts is a promising approach.

**Figure 5:**
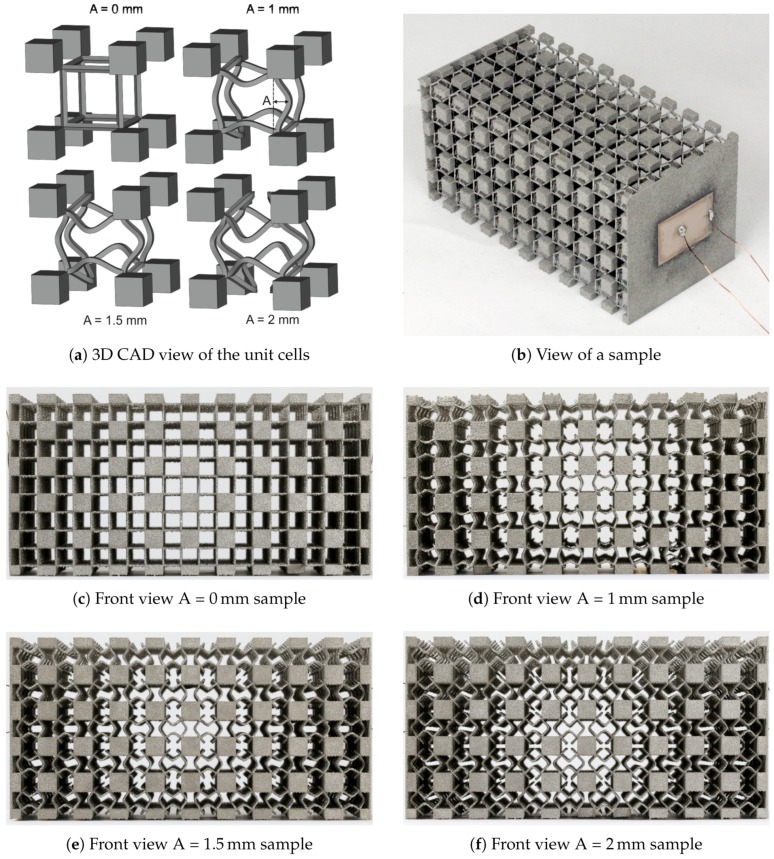
(**a**) CAD view of the four unit cells with Amplitudes of 0 mm, 1 mm, 1.5 mm and 2 mm. The unit cell size is 12 mm; (**b**) Scenographic view of a sample. The piezoceramic actor attached to the surface of the sample is visible; (**c**) Front view of the 5 × 5 × 10 unit cell sample with an amplitude of 0 mm; (**d**) 1 mm; (**e**) 1.5 mm and (**f**) 2 mm.

The unit cell is constructed in CAD (computer aided design) and consists of sinusoidal struts with varying amplitudes in the center surrounded by cubes touching at the node points of the struts (see Figure 5a). The samples are 5 × 5 × 10 periodic arrays of the 12 mm cubic unit cell as depicted in Figure 5b. The node distance of the struts is 6 mm and the cubes have an edge length of 3 mm, which, in turn, results in 6 mm edge lengths in the periodic arrangement. Additionally, on the ends of the long vertices of the array, two thin walls are placed as mounting points for the piezoelectric plates (see transmission measurement setup described in Section 3.2). The samples with the four different amplitudes of 0 mm, 1 mm, 1.5 mm and 2 mm can be seen in Figure 5c–f. Here, the amplitude is defined as the distance between the imaginary line connecting two strut points and the deflected strut as viewed from one of the main directions. Naturally, an amplitude of zero leads to straight struts. The cubes and the sinusoidal struts were built with different parameters during the SEBM process (see Table 1).

The measurement setup is the same as described in [21]. Elastic waves are excited and measured by a piezoelectric actor and sensor, respectively. The sensors/actors are glued on both sides of the sample where a thin wall was built (see Figure 5b). A frequency generator is connected to the piezoelectric actor and creates a sinusoidal wave signal that results in a mechanical deformation in the plate, thus creating elastic waves in the sample. The transmitted signal as well as the incoming signal are measured at the piezoelectric sensor on the opposing side of the sample and at the side where the frequency generator is connected, respectively. For every frequency step, 64 signals are averaged into one and a Gaussian filter is applied to the resulting curve to eliminate sharp peaks from the signal. The magnitude of the transmission *T* is characterized by the maximum voltage amplitude $U_{out}$ at the sensor and the maximum excitation voltage amplitude $U_{in}$ as $T=U_{out}/U_{in}$ for every measured frequency.

## 3. Results

### 3.1. Optimized Unit Cells

The optimization problem (4)–(13) is difficult to solve, especially due to the non-smoothness of the PBG optimization. There are three parameters in the problem, namely, a smoothing parameter β controlling the grayness of the interface, a curvature bound c* for the shape variables, and the minimal profile value $W_{-}$*. The start design for the optimization are always straight orthogonal struts, which do not exhibit a band gap. The FEM discretization is 200×200 with first order quadrilaterals. There is a chance that the optimizer gets stuck in a local optimum and we use the parameters β∈[250,350] and c* ∈[0.02,0.14] similar to a random shots approach. Indeed, we found a common design principle for all parameters. The obtained PBG width depends only on the minimal profile variable $W_{-}$* but is independent on β and c*.

Note that we rescale geometrical and material parameters to improve numerical stability for the optimization. Absolute frequency values for 2D optimization designs are therefore to be considered with care.

**Figure 6:**
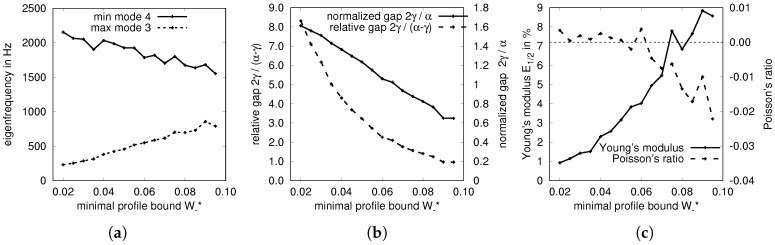
Results of the optimization problem (4)–(13) performed for a wide range of minimal profile widths $W_{-}$* (13). See also Figure 7. (**a**) Absolute band gap; (**b**) Dimensionless band gap; (**c**) Observed properties.

**Figure 7:**
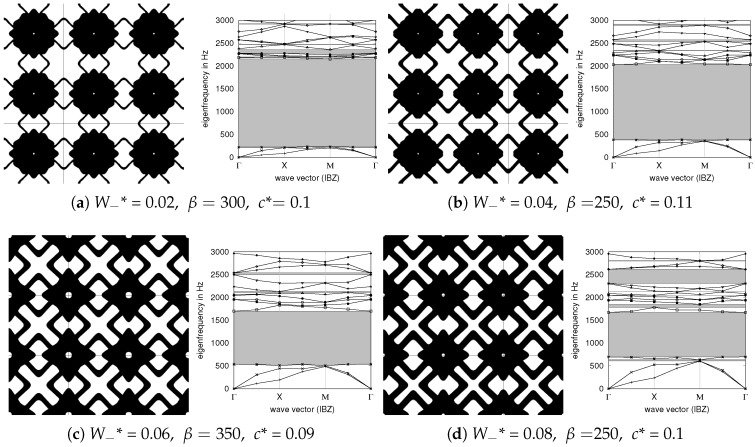
Selected designs obtained by optimization. Note the common design principle. The obtained relative band gaps for (**a**–**d**) are 8.32, 4.30, 2.16 and 1.40, respectively, the normalized band gaps are 1.61, 1.37, 1.04 and 0.85, see also Figure 6b. Additionally, the design for $W_{-}$*=0.085 is shown in Figure 4a. Due to the scaling of the numerical model, the actual frequency values are not comparable to real materials.

In Figure 6a,b the widths of the optimized PBGs are shown with respect to the minimal profile variable bound $W_{-}$*. This corresponds to a minimal bar thickness of $\sqrt{2}\;W_{-}$* and indirectly corresponds with the stiffness of the structure, which is shown in Figure 6c ranging from 1 to 9% of the material’s Young’s modulus. The resulting volume fraction ranges from 50 to 60%. Note that solely maximizing the Young’s modulus of an orthotropic structure results in 33% of the stiffness of solid material for a volume fraction of 60% (2D optimization of material properties can be performed online at http://eamc080.eam.uni-erlangen.de/iTop/). Figure 6c also shows that the resulting Poisson’s ratio is slightly negative for sufficiently large minimal feature size. Note that our shape mapping approach would also allow to add the Young’s modulus and Poisson’s ratio to the problem formulation in terms of constraints or subject to maximization/minimization. Selected designs and their dispersion diagrams are shown in Figure 7.

### 3.2. Transmission Measurements

**Figure 8:**
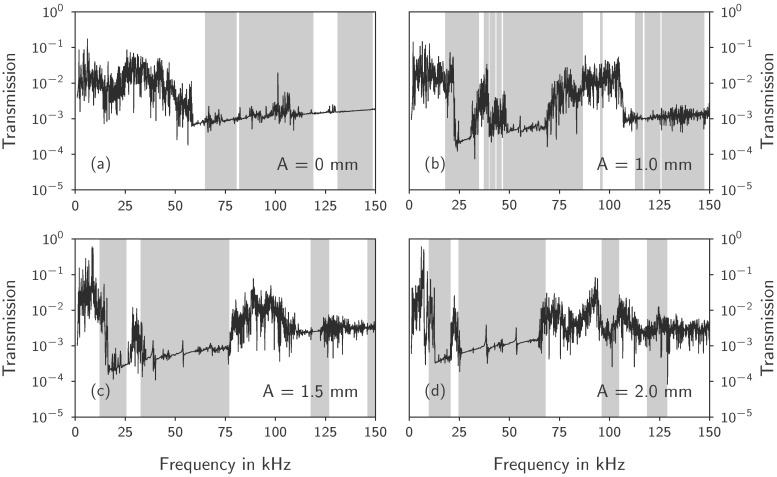
Transmission measurement results for unit cell amplitudes of (**a**) 0 mm; (**b**) 1 mm; (**c**) 1.5 mm and (**d**) 2 mm. PBGs calculated from dispersion relations of the respective unit cells are marked as gray bands. PBGs with widths of less than 1 kHz are omitted.

The results of the transmission measurements are shown in Figure 8. The transmission is only measured along one wave direction (Γ→X), even though the numerical dispersion relations show that the designs exhibit PBG behaviour in arbitrary directions. In general, the frequency response diagrams show a low transmission in areas where PBGs were determined numerically. The gray areas indicate PBGs from the numerical dispersion relations of the 3D unit cells as described in Section 2.1, while frequency ranges of consistently low transmission value indicate the experimentally obtained PBGs. In all samples, a relatively low transmission of 0.01 can be seen in the non-PBG areas. Reasons for the low transmission are the sensitivity of the setup, the adhesive joint of the piezoceramic plates and dampening of the material itself. The sample with straight struts in Figure 8a shows the lower bound of the first measured PBG at 55 kHz to 60 kHz in the transmission plot, while the dispersion relation resulted in the first PBG starting at 65 kHz. The signal is strongly oscillating below the first PBG and remains at a low transmission above. The dispersion relation results indicate a pass band around 125 kHz that is not reflected in the transmission spectrum.

The other three samples show a different behavior than the first. All three samples have pronounced PBGs in a low frequency range inside the audible frequencies (lower PBG boundaries from numerical dispersion relations: 18 kHz 12 kHz 10 kHz for the amplitudes 1.0 mm, 1.5 mm and 2.0 mm, respectively). The transmission diagrams show clear drops in transmission at these frequencies. Following the first PBG, the transmission rises back to a higher level for all three samples. The second PBG of the samples with 1.5 mm and 2 mm amplitude has a width of approximately 40 kHz as can be seen in both transmission diagram and dispersion relation. The 1 mm amplitude sample has three narrow PBGs below the large band gap of almost 40 kHz. While the samples with the largest amplitudes show good agreement of numerical and experimental data in the second PBG, the sample with 1 mm amplitude shows a narrower experimental PBG.

After the second PBG, the sample with 1 mm amplitude behaves differently compared to the other two samples. It has two very narrow PBGs at 95 kHz and 115 kHz. A pass band of only 0.5 kHz is then followed by a large PBG. The experimental frequency response shows transmission in the frequency range of 75kHz to 110 kHz which is wider than the pass band indicated by the numerical results. From 110 kHz the frequency response shows a PBG matching to the numerical results.

The samples with the largest amplitudes only show four PBGs in the considered frequency range. The third and the fourth experimentally determined PBG are not as distinguishable as the first two. Nevertheless, both samples show transmission drops around the PBG frequencies indicated by the numerical data.

## 4. Discussion

We have presented a novel modeling for structural optimization that combines features of parametric shape optimization with density based topology optimization. We have successfully applied this approach for a 2D gradient based optimization of PBG structures. A numerical study was performed with a minimal feature size as parameter. The structures where also evaluated numerically to obtain material properties Young’s modulus and Poisson’s ratio where a slight auxetic behaviour could be observed. PBG structures have been designed in the literature manually by gradient free and gradient based optimization, but usually the corresponding stiffness of the structures is not given. Under the assumption of a reciprocally connection of PBG width and stiffness, the results in literature are therefore difficult to compare. However, the authors believe that the presented results are of unmatched performance. The results reveal a simple, common design principle independent on the parametrization of the optimization problem. Note that we designed our optimization approach intentionally with a rather limited design space to cope with the issues of PBG optimization. Hence, for a different design space competitive alternative designs might exist.

In this work, we successfully transferred the concept of connected masses from the 2D optimization results to a 3D design with connections adapted from earlier studies. Ti-6Al-4V samples were manufactured by selective electron beam melting and tested in a frequency response measurement setup. The obtained frequency response diagrams show reasonable agreement with the numerically obtained PBGs from dispersion relations. The biggest factor for deviations that has to be considered is the difficult-to-measure mechanically active strut diameter of the coarse metal struts. Even computer tomography measurements in the past have shown very high standard deviations in strut diameters, rendering an exact determination impossible [21]. Therefore, the initial estimations for the strut thickness of the samples can be considered a good approximation.

While past studies have shown that cubic lattices with straight struts do not show PBGs [20], the presented structure with straight struts and added masses does have a PBG. This can be attributed to the masses acting as inertial elements connected by the much weaker and more elastic struts. Sinusoidal struts, however, have more favorable PBG properties. In the three samples with sinusoidal struts and added masses, PBGs inside the audible frequency range were achieved, possibly enabling applications in high-frequency noise isolation. For lower frequencies, the structures would have to become increasingly bigger and therefore more unfeasible.

In future work, we will extend the optimization to obtain 3D designs directly. Additionally, we will seek structures with band gaps in the lower audible frequency range without changing the unit cell size.

## Figures and Tables

**Table 1:** Parameters used on the Arcam AB Q10 machine for melting the sinusoidal struts and the cubes attached to the corners of the strut unit cell.

Parameter	Struts	Cubes
Current *I*	5 mA	12 mA
Voltage *V*	60 kV	60 kV
Scan speed $v_{B}$	1.5 m $s^{-1}$	10 m $s^{-1}$
Line energy $E_{L}$	0.2 J ${\mathrm{mm}}^{-1}$	0.072 J ${\mathrm{mm}}^{-1}$
Line offset $L_{off}$	-	100 μm
